# Recent Developments in the Reduction of Oxidative Stress through Antioxidant Polymeric Formulations

**DOI:** 10.3390/pharmaceutics11100505

**Published:** 2019-10-01

**Authors:** Muhammad Shajih Zafar, Alessandra Quarta, Marco Marradi, Andrea Ragusa

**Affiliations:** 1 Department of Engineering for Innovation, University of Salento, via Monteroni, 73100 Lecce, Italy; shajihengr@gmail.com; 2 CNR Nanotec, Institute of Nanotechnology, via Monteroni, 73100 Lecce, Italy; alessandra.quarta@nanotec.cnr.it; 3 Department of Chemistry “Ugo Schiff”, University of Florence, via della Lastruccia 13, Sesto Fiorentino, 50019 Firenze, Italy; marco.marradi@unifi.it

**Keywords:** antioxidant, radicals, ROS, polymer, chitosan, extract, film, hydrogel, nanoparticle

## Abstract

Reactive oxygen and nitrogen species (RONS) are produced endogenously in our body, or introduced through external factors, such as pollution, cigarette smoke, and excessive sunlight exposure. In normal conditions, there is a physiological balance between pro-oxidant species and antioxidant molecules that are able to counteract the detrimental effect of the former. Nevertheless, when this homeostasis is disrupted, the resulting oxidative stress can lead to several pathological conditions, from inflammation to cancer and neurodegenerative diseases. In this review, we report on the recent developments of different polymeric formulations that are able to reduce the oxidative stress, from natural extracts, to films and hydrogels, and finally to nanoparticles (NPs).

## 1. Introduction

Oxidative stress results from the imbalance between pro-oxidant species produced either by our body, i.e., radicals caused by stress, or by exogenous environmental factors, such as pollution or excessive sunlight exposure, and antioxidant biomolecules and enzymes unable to intercept and neutralize the former [1]. Endogenous radicals usually come from reactive oxygen species (ROS), such as the hydroxyl radical (^•^OH), hydrogen peroxide (H_2_O_2_), the superoxide anion (^•^O_2_^−^), and singlet oxygen (^1^O_2_), which during homeostasis are counterbalanced, for example, by melanin, glutathione (GSH), glutathione peroxidase (GPx), superoxide dismutase (SOD), and catalase (CAT) [2].

When this balance lacks or cannot be reached, ROS induce an inflammatory status that, in the long term, can trigger the occurrence of several pathologies, such as cancer, cardiovascular, neurological, or pulmonary diseases, to cite a few [3]. Reactive oxygen and nitrogen species (RONS) also play a significant role in aging and in age-linked diseases [4]. The age-related redox inequity is most probably activated by the clear effect of low anti-oxidative resistance systems and augmented fabrication of free radicals [5,6]. Nevertheless, a healthy lifestyle and diet, together with steady workout training, can help to reach equilibrium between oxidative stress and the antioxidant structure [7]. Unfortunately, these precautions might not be enough in situations in which there are already other factors, i.e., genetics, that can trigger a particular disease. In this case, oxidative stress can accelerate the degenerative process. Oxidative stress is a chief reason for tissue damage in brain and also in chronic inflammatory, vascular, and neurodegenerative disorders of the central nervous system [3]. It is pursued through the assembly of responsive oxygen and nitric oxide classes principally by microglia cells and macrophages [8]. The oxidative stress triggers inflammatory processes prominent to the alteration of an ordinary cell to tumor cell, as well as tumor cell proliferation, chemoresistance, radio resistance, invasion, and angiogenesis [9]. ROS signaling is a chief reason in the development and progression of the malignancy [10]. Oxidative stress was also shown to play an important role in the progression of diabetes complications, in both microvascular and cardiovascular ones [11]. Apparently, the metabolic irregularities of diabetes cause an overproduction of mitochondrial superoxide radicals in endothelial cells of both big and small blood vessels and in the myocardium.

In order to alleviate and prevent situations of oxidative stress, many people take antioxidant supplements orally, containing mostly vitamins. However, the efficacy of this treatment has been often criticized because of the low bioavailability of molecules, which really exerts a therapeutic effect [12,13,14]. In fact, most of the vitamins are degraded once ingested, or they do not reach the desired target because of unfavorable biodistribution or absorption limits [15]. Oral drug bioavailability is a multifaceted process linking solubility, permeability, stability into the stomach, and efflux/influx carriers, and all these barriers must be taken into account when delivering a drug, especially if it is very reactive, such as antioxidant molecules [16]. In this regard, polymeric materials can preserve the scavenging properties of the single antioxidant molecules, thus increasing their stability and bioavailability. Also, nanotechnology can give a huge hand in overcoming such hurdles by maximizing the therapeutic efficacy while minimizing the side effects [17]. Nanoparticles (NPs) can protect antioxidant molecules until the desired target site is reached or encapsulate molecules that are poorly soluble in biological media. Similarly, antioxidant hydrogels can directly act on the skin and release in a controlled and sustained way their beneficial effect (e.g., for wound healing). On the other hand, polymeric films with antioxidant properties can be exploited as biocomposites or skin masks, as well as for retaining longer the nutraceutical properties of food products, thus improving their shelf life. Nevertheless, as a common denominator, biotechnological formulations can bring several advantages in the reduction of oxidative stress. The choice of one formulation over another usually depends on the final pharmacological application, and quite often, the manufacturing procedures can be modified and adapted to obtain different formulations from the same polymer, being the antioxidant property intrinsically related to itself.

In this review, we report the most recent polymers with antioxidant properties and analyze the strategies used for delivering them in order to reduce the oxidative stress. The strength of the polymeric antioxidants is usually quantified in vitro by measuring either their efficacy in quenching a known concentration of preformed radicals, typically hydroxyl (OH), 2,2-diphenyl-1-picrylhydrazyl (DPPH), and 2,2′-azino-bis(3-ethylbenzothiazoline-6-sulfonic acid) (ABTS) radical cation, or the enzymatic activity of SOD, GPx, and CAT. Nevertheless, in vivo studies have been also reported. The formulations have been categorized according to four main groups, i.e., antioxidant polymers extracted from natural sources, polymers assembled in films or hydrogels, and NPs able to reduce the oxidative stress. This classification often resembles the type of potential application of the polymeric form, although some exceptions are present. Final considerations about the actual situation and potential future directions are also present.

## 2. Polymeric Formulations

**Figure 1:**
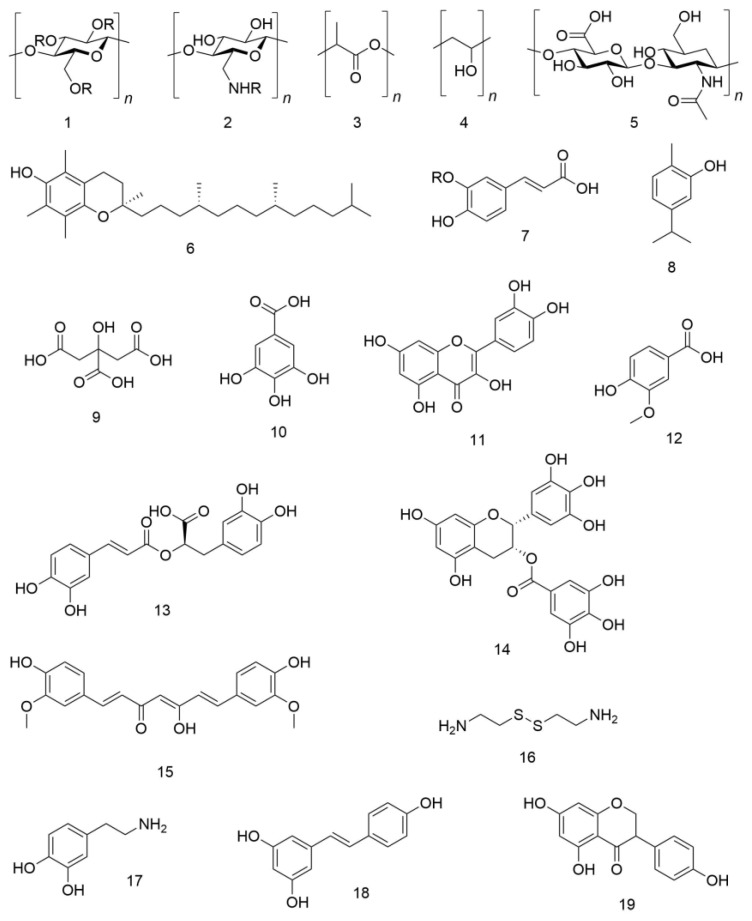
Chemical structures of the main compounds exploited to reduce the oxidative stress, either alone, grafted onto a polymer, or encapsulated into NPs. (**1**) R = H, cellulose; R = CH_2_COOH, carboxymethyl cellulose; (**2**) R = either H or COCH_3_ depending on the degree of deacetylation, chitosan; (**3**) polylactic acid; (**4**) polyvinyl alcohol; (**5**) hyaluronic acid; (**6**) α−tocopherol; (**7**) R = H, caffeic acid; R = CH_3_, ferulic acid; (**8**) carvacrol; (**9**) citric acid; (**10**) gallic acid; (**11**) quercetin; (**12**) vanillic acid; (**13**) rosmarinic acid; (**14**) epigallocatechin gallate; (**15**) curcumin; (**16**) cystamine; (**17**) dopamine; (**18**) resveratrol; and (**19**) genistein.

Polymeric materials have been studied over time because of their abundance, Food and Drug Administration (FDA) and European Medicines Agency (EMA) approval in the market, easy functionalization, and biocompatibility. They have been exploited for a variety of applications, and in the pharmaceutical area, they are mainly represented by polyphenols and polysaccharides, or their combination. Other small antioxidant molecules have also been polymerized to obtain novel biomaterials with stronger antioxidant activity or to exploit the potentiality of the macrostructure. Conjugated structures, either natural or synthetic, have been also tested in order to improve the final efficacy of the biopolymer. The chemical structures of the main compounds exploited to reduce the oxidative stress, either alone, grafted onto a polymer, or encapsulated into NPs, have been reported in Figure 1.

**Figure 2:**
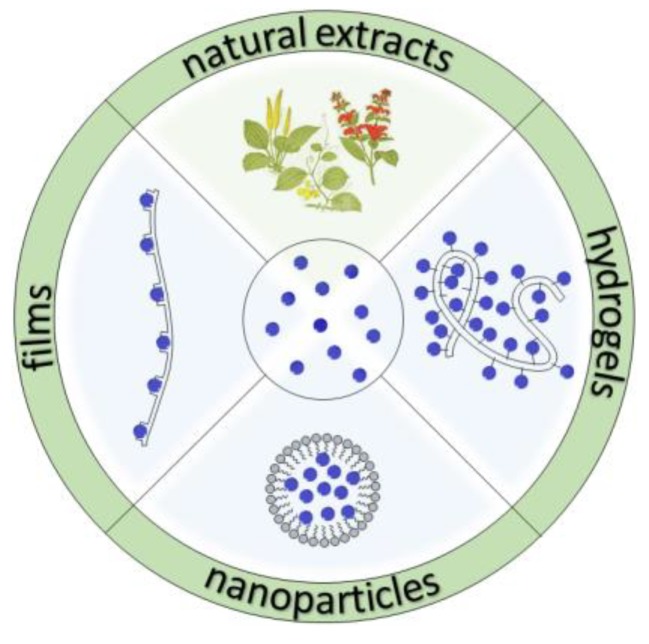
Schematic drawing of antioxidant molecules (central circle) extracted from natural sources (top panel) and exploited for preparing films (left panel), hydrogels (right panel), and nanoparticles (bottom panel) able to reduce the oxidative stress.

Figure 2 reported a schematic representation of the small antioxidant molecules (in the central circle) that have been extracted from natural sources (top panel), and that can be exploited to prepare polymeric films (left panel), hydrogels (right panel), and nanoparticles (bottom panel) depending on the desired application. These categories are now discussed more in detail.

### 2.1. Natural Extracts

The most simple and straightforward way to obtain antioxidant polymers is just by simply extracting them from plants (see Table 1 for a summary of the examples reported here).

These polymers usually have the advantage of being nontoxic, at the employed concentration, and available in large amounts, not considering the absence of any technological sophistication for functionalization and purification, i.e., they are “green”. On the other hand, they have a quite broad molecular weight (MW) distribution and they might vary from batch to batch because of the intrinsic variability of the source, i.e., the plants.

Polymeric materials extracted from natural sources exhibit a wide variety of properties, including antioxidant ones [18,19,20]. For example, polymeric materials with antioxidant properties were extracted from *Astragalus membranaceus* (AME), *Glycyrrhiza uralensis* (GU), and their combination (AG) by Li and colleagues [21]. Interestingly, they found that the ethyl acetate extract of the AG herb pair presented significantly higher antioxidant capacity in vitro than the theoretical sum of two individual herbs, which was probably because of a synergistic effect of the contained phenolic/flavonoid fractions. They also observed higher antioxidant enzymatic activity and better cytoprotection, and the strength of this extract was related to the high concentration of phenols and flavonoids.

Despite phenol derivatives representing a big portion of natural antioxidant compounds, part of their strength is also due to polysaccharides that are able to reduce the oxidative stress because of the numerous hydroxylic groups. For example, several water-extracted fractions from *Phyllanthus Emblica*, a medicinal plant used in traditional Ayurvedic medicine, showed to have antioxidant capacities comparable to butylated hydroxy anisole (BHA) and butylated hydroxy toluene (BHT) used as standards [22]. Nevertheless, the activities of the fractions were proportional to the phenol content, and the major fraction had 50% polysaccharides and 26% phenols. More recently, Khanna and colleagues showed that oral administration of CAPROS^®^, a standardized aqueous extract of the edible fruit of the same plant containing about 60% of low MW hydrolysable tannins such as emblicanin-A, emblicanin-B, punigluconin, and pedunculagin, induced a decrease of several cardiovascular disease (CVD) risk factors by two-thirds after 12 weeks [23]. A polysaccharide extracted from *Acanthophyllum acerosum* roots, comprised of 20.8% d-glucose, 66.2% d-galactose, and 13.0% l-arabinose, was also able to scavenge DPPH radicals, but its activity was less than that of ascorbic acid at the same concentration [24].

*Epimedium* is another medicinal herb, although of Chinese tradition, comprising many species well known for their antioxidant properties, which are also related to the high content of flavonoids [25]. Polysaccharides extracted by hot water from *Epimedium acuminatum*, despite having a similar chemical composition with significant differences only for the uronic acid content, exhibited about 50% higher antioxidant activities than those obtained by other extraction processes, such as ultrasonication, enzymatic, and microwave-assisted extraction, apparently because of a more regular and smoother surface, indicating that the morphology of the final biomaterial also plays a significant role on its final properties [26].

*Pharbitis nil* Choisy (PN, also known as Japanese morning glory) is an ornamental tropical plant used in Chinese herbal medicine [27]. Wang et al. optimized an ultrasonication method for extracting from its seeds mainly polysaccharides, with minor percentages of uronic acid and proteins, which showed remarkable ABTS and DPPH radical scavenging activities [28]. On the other hand, Shu and colleagues used an enzymatic method (cellulase-assisted) for extracting polysaccharides from white hyacinth bean [29]. Despite the lower scavenging activity compared to ascorbic acid, which was used as a reference standard at the same concentration, the polysaccharides were also able to stimulate the growth of several probiotics.

β-Glucans are polysaccharides with strong immunostimulating effects that influence cytokine production and antibody response [30]. β-Glucan extracted from barley, which mostly comprises β-(1,3-1,4)-d-glucan, was shown to possess antioxidant activity that varied depending on its structure and molecular weight, i.e., from the source and the extraction method [31]. Nevertheless, the capacity to reduce oxidative stress was significantly higher than that of several polymers used as food additives (i.e., about 50% compared to pectin and more than 60% compared to chitosan), supposedly because the β-glucan decreased the number of pro-inflammatory cytokines (mostly IL-6 and TNF-α), and increased that of the antioxidants. Laminaran is a small glucan extracted from brown seaweeds with chains that are either ended by d-mannitol residues (M-series) or by d-glucose residues (G-series). Sellimi et al. prepared a cream based on laminaran polysaccharide extracted from *Cystoseira barbata* that showed noticeable antimicrobial and antioxidant properties in vitro, as well as wound-healing promotion in vivo [32].

In addition to polysaccharides, other types of biomolecules have been also exploited for reducing oxidative stress. One of these is c-phycocyanin (c-PC), which is a water-soluble pigment present in cyanobacteria and red algae, such as the *Spirulina*, that is well-known for its nutritional and therapeutic value [33,34,35]. The radical scavenging ability of c-PC has been related to its tetrapyrrole chromophore phycocyanobilin, which apparently chelates iron ions and inhibits deoxyribose degradation with an IC_50_ value of 13 μM. Pleonsil et al. prepared a recombinant apo-c-PC β subunit from a cloned gene expressed in *Escherichia coli*; nevertheless, its antioxidant activity, despite being still present to some extent, was lower than that of the natural extract, confirming the scavenging role of the bilin chromophore [36]. On the other hand, Wu and colleagues biosynthesized a PCB-CpcB(C-82) fluorescent phycocyanin β subunit from *Spirulina subsalsa* that showed stronger hydroxyl and DPPH free radicals scavenging activity than apo-CpcB, probably because of the bilin binding [37].

c-PC extracts were also exploited to prevent the H_2_O_2_-induced impairment of mitochondrial membrane potential, the release of cytochrome c from the mitochondria, and ROS generation in porcine embryos [38]. In addition, c-PC was able to reduce apoptosis, DNA damage, and autophagy in the oxidatively stressed blastocysts. Finally, Park et al. quantified the two major radical scavenging components of several commercially available *Spirulina* powders, i.e., carotenoids and c-PC, and correlated their color (orange and blue, respectively) to the antioxidant activity through DPPH and ABTS assays, demonstrating that the c-PC extract had a stronger antioxidant activity compared to the carotenoid fraction [39].

### 2.2. Films

Polymeric films have attracted much interest because of the easy functionalization of their surface with several chemical groups, thus yielding highly versatile biomaterials whose properties can be tuned according to the final biomedical application, such as biocompatible coatings for implants and thin films for tissue engineering or for gene therapy and drug delivery, i.e., in wound healing. Free radicals can also induce lipid peroxidation and, in addition to the biomedical consequences in human body, they affect negatively the quality, safety, and shelf life of food products [40]. Antioxidant supplementation as food additives can slow down degenerative processes, thus preserving flavor and nutritional values. A complementary approach exploits the use of polymeric films with antioxidant properties for protecting nutraceuticals from degradation, especially when the use of synthetic antioxidants has been restricted because of their potential carcinogenic effects. In this direction, the use of biocompatible natural polymers, such as polysaccharides, minimizes the risk of safety issues. Furthermore, functionalization of the polymer with antioxidant molecules allows obtaining novel biomaterials with improved radical scavenging ability (see Table 2 for a summary of the examples reported here). 

Chitosan is well-known for its antioxidant properties, especially when at low MW and with a high degree of deacetylation [41,42]. Hromis et al. synthetized a chitosan film integrated with four oleoresins: garlic, black pepper, caraway, and cinnamon as packaging material for food preservation [43]. This chitosan film with oleoresins showed good barrier properties to oxygen and air, but less sensitivity to moisture with respect to pure chitosan film. Chitosan films were also functionalized with montmorillonite (MMT) and pomegranate rind powder extract (PRP), which are rich in polyphenolic compounds such as ellagic acid, ellagic tannins, and gallic acid (GA) [44]. Due to the two additional components, the authors were able to obtain a material with enhanced water vapor permeability and mechanical properties of the chitosan-based films (thanks to the MMT) and excellent antioxidant activities (because of the PRP). On the other hand, Yuan and colleagues synthetized chitosan-based films incorporating carvacrol, pomegranate peel extract (PPE), and carvacrol + PPE, and discovered that all three combinations decreased the transparency of the unfunctionalized chitosan film, but improved antioxidant activity [45]. Furthermore, the film incorporating carvacrol and PPE also exhibited good antibacterial activity against *Staphylococcus aureus* and *Escherichia coli*.

The combination of chitosan with caffeic acid (CA) or GA using laccase from *Trametes versicolor* yielded a product with better antioxidant and antimicrobial properties that could be modulated by varying the pH, with the best activity at pH 4.5 [46]. Chitosan was also functionalized with the lipophilic α-tocopherol, which is a type of vitamin E usually found in oils extracted from several plants [47]. Martins and colleagues found that α-tocopherol increased the film opacity, thus offering improved UV protection, as well as higher water vapor permeability and better antioxidant capacity, thus enhancing the overall quality of the chitosan film as a food packaging biomaterial.

The combination of several oil and water-based natural antioxidants into the chitosan films was performed to estimate the antioxidant and physical properties of the resulting polymers [48]. Five essential oils (EO) (rosemary, ginger, sage, tea tree, and thyme EO) and six diverse hydroalcoholic extracts (HAE) (ginger, rosemary, sage, black tea, green tea, and kenaf leaves) were introduced, yielding chitosan films with higher antioxidant activities. EO and even more HAE conferred to chitosan an extra protection against oxidative processes by improving the light barrier of the films. Among all the extracts, black tea and green tea from HAE and sage, thyme, and rosemary from EO were the most promising antioxidants also because of the improved tensile strength of the prepared films. The authors later investigated the migration of the antioxidants from the prepared films and observed that those incorporating ginger, sage, or rosemary EO showed the highest diffusion and antioxidant activity [49]. Cao et al. synthetized a biofilm from chitosan and inulin, which is a polysaccharide extracted from burdock root, also incorporating oregano and thyme essential oils [50]. Films prepared with both polymers showed better physicochemical properties, such as increased textile strength and elongation to break, compared to those without chitosan. Furthermore, all films incorporating EO showed antioxidant and antimicrobial activity, with 2% EO giving the best DPPH scavenging results.

Annatto (*Bixa orellana*) seeds powder is a nontoxic natural dye with a yellow–orange–red color because of the high value of carotenoids, which are mostly bixin and norbixin, with interesting biological properties, such as antioxidant and free radical scavenging activities as well as antibacterial, anti-inflammatory, and neuropharmacological abilities [51]. The incorporation of annatto powder and vitamin C into reacetylated chitosan (degree of acetylation 33.6%) films improved significantly the ROS scavenging ability, by releasing the antioxidant compounds, and could find potential application as an anti-aging skin mask [52].

Starch is one of the most abundant natural polysaccharides, mainly composed of amylose and amylopectin, and it has attracted considerable attention also as a biodegradable thermoplastic polymer [53]. Edible films were developed from chitosan and wheat starch using glycerol as a plasticizer and four active components as antioxidants, namely citric acid, α-tocopherol, thyme, and basil essential oil [54]. The mechanical properties of the films were barely affected by the antioxidants, but those containing α-tocopherol showed higher antioxidant ability. Kim et al. incorporated various amounts of cocoa nibs extract (CNE, 0.3%, 0.7%, and 1%) into starch extracted from adzuki bean [55]. As expected, the scavenging activity was proportional to the antioxidant concentration, with the 1% CNE-containing film able to quench 100% of ABTS and 94% of DPPH produced radicals.

Recently, much interest has been devoted to the reutilization of crop wastes. Among them, lignin extracted from agricultural residues is gaining an ever-increasing attention as an antioxidant because of its phenolic structure, with many potential applications in healthcare and agriculture [56]. Arshanitsa et al. investigated the lignin fractions obtained from the extraction with solvents of different polarity and correlated their antioxidant activity in polyurethane films to their structure [57]. In this way, they found a reproducible method for obtaining homogeneous lignin products with reliable physicochemical properties that are suitable for industrial processes and applications. On the other hand, cellulose nanofibrils were exploited for preparing biodegradable films in conjunction with tannin extract [58]. The best results were obtained by adding 5% (*w/w*) extract, thus obtaining multi-functional hybrid films with improved antioxidant and UV-adsorbing properties, and potential applications as biocomposites and packaging materials. Carboxymethyl cellulose (CMC) was also combined with sodium alginate (SA) and epigallocatechin gallate (EGCG) for fabricating active edible films slowly releasing EGCG that showed strong antioxidant activity in fatty foods [59].

Despite polysaccharides being among the most exploited polymers because of their abundance, cheapness, and biocompatibility, other materials have also been used for preparing films with antioxidant capacity. For example, Zhai and colleagues condensed tannin from larch bark with polyvinyl alcohol (PVA) [60]. DPPH assay and starch–potassium iodide oxidation–discoloration analysis showed that the composite membranes have good antioxidative activities. On the other hand, Dintcheva et al. utilized various amounts of natural phenolic compounds, such as ferulic acid (FA), vanillic acid (VA), vitamin E (VE), and quercetin (Q), for preparing PLA films [61]. Among these natural phenolic compounds, FA and Q showed the highest antioxidant properties when combined at low concentration with PLA, while at higher concentrations, they exerted a pronounced prodegradant action to the PLA matrix.

Rosmarinic acid (RosA), a naturally water-soluble phenolic compound, was incorporated onto a gelatin backbone, and then glycerol was used as a plasticizer, and the matrix was cross-linked by dialdehyde xanthan gum (DXG) to produce active gelatin-based edible films [62]. The obtained RosA-gelatin edible film possessed excellent ultraviolet barrier capacity and exhibited good antioxidant properties and long-term antibacterial activity, with promising applications in the fields of food and pharmaceutical packaging. Garcia-Orue et al. developed an elegant gelatin-based bilayer suitable for wound healing [63]. They first prepared a lactose-based gelatin, for mechanical support and protection, and then cross-linked a lower layer of citric acid and chitosan for antioxidant and swelling properties. The hydrofilm was tested by ex vivo assay in human skin showing to be biocompatible and allowing the healing process. Liang and Wang incorporated into a soybean protein isolate different concentrations of cortex *Phellodendron* extract (CPE) obtaining an active film with good rheological properties, but additional antioxidant and antimicrobial properties against *Staphylococcus aureus* bacteria (the best compromise being 15% CPE *w/w*), which is potentially useful for improving food products’ shelf life [64].

### 2.3. Hydrogels

When polymeric materials entrap large amounts of water inside their matrix, a hydrogel is formed. This type of material is used for a variety of applications, including pharmaceutical and biomedical ones, such as drug delivery, wound dressing, and tissue engineering, to cite a few [65] (see Table 3 for the examples reported here). 

Poly(antioxidant *β*-amino ester) (PAbAE) biodegradable hydrogels with two polyphenolic antioxidants, i.e., curcumin and quercetin, were prepared in a two-step polymerization process by Wattamwar et al. [66]. The degradation rate of the hydrogels could be controlled by the monomers used during the synthesis. Furthermore, PAbAE degradation products suppressed the hydrogen peroxide-induced oxidative stress in human umbilical vein endothelial (HUVEC) cells, impeding ROS-induced cell death and maintaining cellular viability. They also set up a single-phase process for preparing quercetin-based PAbAE gels (25–38 wt% loading) that were hydrolyzed over 48 h, slowly releasing the antioxidant and inhibiting the oxidative stress [67]. Similar curcumin-based PAbAE gels were also able to protect cells from radicals, but in addition, they increased the tolerated cytotoxic curcumin concentration as compared to the free molecule [68]. On the other hand, PAbAE hydrogels with redox-sensitive disulfide (cystamine) cross-linking were able to sense the redox state of the environment, thus changing their degradation products, and finally increasing the IC_50_ of the material by an order of magnitude [69].

Conductive injectable self-healed hydrogels were prepared by using quaternized chitosan-g-polyaniline (QCSP) and benzaldehyde group functionalized poly(ethylene glycol)-*co*-poly(glycerol sebacate) as antibacterial, antioxidant, and electroactive dressing [70]. These hydrogels offered good self-healing, free radical scavenging ability, antibacterial, and antioxidant activities for cutaneous wound-healing applications, and the best candidate also showed excellent in vivo blood clotting capacity and an enhanced in vivo wound-healing process.

Qu et al. mixed *N*-carboxyethyl chitosan (CEC) with hyaluronic acid-graft-aniline tetramer (OHA-AT) polymer in physiological conditions obtaining electroactive injectable OHA-AT/CEC hydrogels that were shown to have a high free radical scavenging capacity, high swelling ratio, and antimicrobial property with auspicious applications in wound dressing [71]. The encapsulation of amoxicillin into the hydrogel also conferred antibacterial properties for preventing wound infection. In vivo experiments showed that the OHA-AT/CEC hydrogel effectively accelerated the healing process.

Sahiner and colleagues synthetized a bulk poly(tannic acid) (pTA) hydrogel by cross-linking TA molecules with a trimethylolpropane triglycidyl ether epoxy cross-linker with good antioxidant ability, especially at slightly acidic pH, probably because of the TA hydrolysis to release gallic acid [72]. The degraded p(TA) hydrogels also showed robust antimicrobial property against Gram-positive (*Staphylococcus aureus* and *Bacillus subtilis*) and Gram-negative (*Pseudomonas aeruginosa*) bacteria, as well as against the *Candida albicans* fungus strain. TA was also used by Lee et al. for cross-linking hyaluronic acid (HA) hydrogels to slow down their usually rapid degradation process under physiological conditions [73]. The enzymatic degradation of the resulting HA-TA hydrogel was significantly improved, while retaining the antioxidant capacity of the polyphenol.

Polyphenol-modified (gallic acid and dopamine) chitosan hydrogels were also prepared by Kim and colleagues [74,75]. The resulting antioxidant capacity was stronger in the GA-functionalized hydrogels, supposedly because of the higher number of hydroxylic groups, and in the hydrogels prepared with longer chitosan chains, probably because of the higher number of conjugable polyphenols.

Gupta et al. designed and synthetized an elegant ABC triblock polymer (poly[(propylenesulfide)-*block*-(*N*,*N*-dimethylacrylamide)-*block*-(*N*-isopropylacrylamide)], PPS-*b*-PDMA-*b*-PNIPAAM) with thermoresponsive shape modification at physiological temperature (from micelles to hydrogel), and ROS triggered degradation and drug release once exposed to oxidative stress [76]. The properties of the triblock hydrogel could be tuned by varying the polymeric composition, thus obtaining in vivo differential release kinetics according to the specific degradation mechanism [77]. Thermosensitive polypeptide hydrogels based on methoxy poly(ethylene glycol)-poly(l-methionine) diblock copolymers were also prepared by Xu et al. [78]. The release profile of Rhodamine 6G was accelerated under oxidative stress conditions, both in vitro and in vivo. Nevertheless, the hydrogel maintained good biocompatibility in rats, and it was completely degraded over six weeks after subcutaneous injection.

Cerium oxide nanoparticles (CONPs) have the potential to provide broad free radical protection and have the unique ability to switch their oxidative states between III and IV, thus regenerating themselves [79]. CONPs were encapsulated in an alginate hydrogel, and the resulting composite demonstrated dose-dependent protection to beta cells from superoxide exposure with negligible cytotoxicity at a nanoparticle concentration that was 10-fold higher than free CONPs [80].

### 2.4. Nanoparticles

One of the problems of conventional delivery methods is the poor efficacy of the antioxidant when administered orally, because of the partial degradation and the low amount of compound that reaches the target site. This last issue is not relevant when the oxidative stress has to be reduced superficially, i.e., on the skin, in which case films and hydrogels can partially solve the problem thanks to the local administration. On the other hand, nanotechnology can help to override more traditional issues, as it has already done in many biological research areas in the last decades (see Table 4 for the examples reported here) [81,82,83]. 

Ideally, polymeric NPs can encapsulate poorly soluble molecules, protect them from degradation, and release them once they reach the target site [84]. Alternatively, they can be simply prepared with polymeric materials already possessing radical scavenging capacity. For example, melanin-like NPs were synthesized by simple spontaneous air oxidation of the neurotransmitter dopamine under basic conditions [85]. The obtained polyethylene glycole (PEG)-coated sub-100 nm melanin-like NPs exhibited free radical scavenging capacity similar to that of ascorbic acid by DPPH assay and good viability under the same conditions with HeLa cells. Zhao and colleagues demonstrated the capacity of polydopamine (pDA) NPs to reduce ROS levels in vivo in murine macrophages challenged with either H_2_O_2_ or lipopolysaccharides (LPS), as well as their ability to alleviate both acute peritonitis and acute lung injury inflammation in murine models [86]. On the other hand, Wang and colleagues synthesized pDA-coated hemoglobin (Hb) NPs able to reduce efficiently the intracellular oxidative stress without affecting the blood constituents [87]. Very recently, Bao et al. exploited pDA NPs to efficiently reduce ROS-induced inflammation in vivo by subgingival injection in a murine periodontitis model with minimal toxicity [88]. Liu et al. investigated in detail the multi-antioxidative mechanism of action of this type of NPs, demonstrating their activity against multiple RONS, such as O_2_^•–^, H_2_O_2_, ^•^OH, ^•^NO, and ONOO^–^, and how they could be exploited in a rat model of ischemic stroke [89]. Despite pDA NPs being widely investigated, the delivery of simple dopamine molecules was also achieved by Malvindi et al. through an enzyme-responsive theranostic system able to target GLUT-1, a glucose transporter localized in the mammalian blood–brain barrier (BBB) [90].

Aside from catecholamines and their derivatives, other types of polymers have been also shown to be able to scavenge free radicals. A fully biodegradable hydroxybenzyl alcohol (HBA)-incorporated polyoxalate (HPOX) polymer was fabricated and employed as a novel therapeutic for treating airway inflammatory diseases [91]. HPOX NPs were designed to integrate the anti-oxidant and anti-inflammatory properties of HBA and the peroxalate ester linkage’s ability to react to H_2_O_2_ and they showed an ability to suppress the expression of pro-inflammatory mediators such as inducible nitric oxide synthase (iNOS) and interleukin (IL)-4 in a murine model of asthma. Jeong et al. developed biodegradable vanillyl alcohol-containing copolyoxalate (PVAX) microparticles encapsulating dexamethasone (DEX) [92]. DEX-loaded PVAX microparticles worked in a synergistic way, significantly reducing the oxidative stress and suppressing the expression of pro-inflammatory TNF-α and iNOS in the lung of ovalbumin-challenged asthmatic mice without inducing any inflammatory response to the lung tissues. A synergistic effect was also noted by Larrañaga et al., who prepared polymeric capsules loaded with CAT and functionalized with an external layer of TA through a layer-by-layer method using calcium carbonate as a sacrificial model [93]. These capsules were able to efficiently inhibit the oxidative stress and to prevent the expression of matrix metalloproteinase-3 (MMP-3), disintegrin, and metalloproteinase with thrombospondin motif-5 (ADAMTS-5) in an in vitro inflammation model of degenerative disc disease.

Polymeric NPs based on smart synthetic amphiphilic copolymers were used to transport and release in a controlled way dexamethasone in the inner ear to protect against the ototoxic effect of cisplatin [94]. Polymers based on the methacrylic derivatives of ibuprofen, α-tocopherol, and α-tocopheryl succinate were connected by free polymerization to vinylimidazole and vinylpyrrolidone, and then precipitated to obtain the pH-responsive NPs. In vitro biological tests showed lower toxicity induced by cisplatin, the downregulation of caspase 3/7 expression, and lower IL-1β release and intracellular ROS accumulation, while in vivo murine experiments demonstrated a reduced hearing loss when animals were treated with the NPs.

The antioxidant properties of chitosan have been widely investigated, and their use in other formulations has already been described in the previous sections. Chitosan NPs exploit the antioxidant ability of the polymer with novel nanotechnological approaches to further extend its capabilities. Chitosan NPs encapsulating dopamine were shown to significantly reduce the oxidative stress in SHSY-5Y cells, as revealed by the increased enzymatic activity of both GPx and SOD enzymes in averting oxidative stress [95]. Chitosan NPs loading genistein were prepared for overcoming the poor water solubility and rapid clearance of the natural extract, obtaining an efficient nanocarrier that was able to potentially deliver the drug to the brain after permeation through the nasal mucosa [96]. Ray et al. encapsulated multiple antioxidants into chitosan NPs, namely curcumin (Cur), quercetin (Quer), and aspirin (Asp) [97]. The simultaneous delivery resulted in a synergistic effect in inhibiting colon cancer progression in HCT-116 cells as compared to each single treatment, probably because of the mucoadhesive properties of chitosan, which can improve the bioavailability of the drugs, and the different pathways jointly activated by the drugs. On the other hand, Friedrich et al. combined curcumin with resveratrol into lipid-core nanocapsules, thus increasing the photostability of the two polyphenols, and exploited the formulation for skin disease treatment [98]. Co-administration of the two drugs provided a sustained release while facilitating skin absorption and allowing a deeper penetration of resveratrol compared to the individual component. Resveratrol was also encapsulated together with DAP5, a *N*-methyl-D-aspartate (NMDA) receptor inhibitor, into poly(*N*-vinylpyrrolidone)-block-poly-*ε*-caprolactone (PVP-*b*-PCL) copolymer to obtain 200-nm NPs that were able to decrease the production of pro-inflammatory cytokines and attenuate renal I/R injury in vivo, as also confirmed by increased cell viability and SOD level [99]. On the other hand, Caddeo and colleagues prepared PEG-modified liposome releasing resveratrol that inhibited DPPH radicals almost completely (∼93%) in vitro, and that were also able to ensure an optimal protection (>90%) against oxidative stress ex vivo in human erythrocytes [100].

Curcumin was also encapsulated into liposomes by the micelle-to-vesicle transition method and coated with a pH-responsive polymer (Eudragit S100) [101]. The ABTS antioxidant activity of the curcumin-loaded liposomes was comparable to that of free curcumin, and the addition of a polymeric coating was shown to inhibit the release of the antioxidant until its degradation. Tiwari and colleagues synthesized curcumin-loaded poly(lactic-*co*-glycolic acid) (PLGA) NPs, and demonstrated that they could induce neural stem cells proliferation and neuronal differentiation in the hippocampus and subventricular zone of adult rats, as compared to uncoated bulk curcumin, with potential applications in neurodegenerative diseases by stimulating a brain self-repair mechanism [102]. Pure curcumin NPs (230–240 nm) were also prepared by solution-enhanced dispersion via supercritical CO_2_ (SEDS) and displayed DPPH scavenging efficiency almost comparable to that of ascorbic acid (at 2000 μg/mL), representing a valid alternative to traditional curcumin delivery methods [103].

An aqueous extract from *Syzygium cumini* seeds was encapsulated into the biocompatible PCL polymer, and the antioxidant properties of the extract were shown to be preserved by scavenging of DPPH radicals and by the ferric reducing antioxidant power assay (FRAP) [104]. Additionally, the NPs displayed high protection against oxidized LDL particles in vitro, as well as antifungal activity against *Candida guilliermondii* and *Candida haemulonii*, without any appreciable toxicity. PCL NPs were also exploited for the incorporation of *Ilex paraguariensis* extract, significantly reducing the quantity of chlorogenic acid permeated through the skin (up to 12 h ex vivo), thus extending its topical antioxidant effect [105].

Carbon nanoparticles (CNPs) derived by glucose have been also reported to have pro- or anti-oxidant properties [106]. Kokalari and colleagues demonstrated that when irradiated with a near-infrared (NIR) laser, CNPs generate heat and singlet oxygen (^1^O_2_), which is useful for dual photothermal (PT)/photodynamic (PD) therapy in cancer treatment [107]. On the other hand, CNPs react with both oxidant (hydroxyl radicals) and antioxidant (glutathione) species when not photoactivated in a cell-free system. However, in macrophages, the antioxidant effect is predominant, thus suggesting a potential protective effect from the oxidative stress induced by activated macrophages.

Polymer-coated inorganic NPs have been also used for reducing the oxidative stress by exploiting the property of metal ions with multiple redox state that are known for scavenging free radicals. For example, Ni et al. developed molybdenum-based polyoxometalate (POM) nanoclusters that were able to scavenge detrimental ROS, thus efficiently alleviating clinical symptoms in mice affected by acute kidney injury [108].

Selenium is an essential trace element present in the human body with an antioxidant role, and it can be found in several multivitamin dietary supplements. Li et al. prepared selenium-doped carbon quantum dots that were able to reduce the oxidative stress in MDA-MB-231 cells and with redox-dependent reversible green fluorescence properties [109]. On the other hand, Zhai and colleagues found out that low MW chitosan-coated selenium (CS-Se) NPs could efficiently penetrate mice tissues and protect GPx activity induced by UV radiation [110]. Interestingly, the antioxidant capacities of the CS-Se NPs were more evident in viscera than in skin. Biogenic selenium NPs were obtained from *Z. Officinale* root extract biomolecules, which acted both as reducing agents as well as stabilizing agents [111]. These NPs showed good antimicrobial activity and excellent radical scavenging activity when compared to that of ascorbic acid, as measured by DPPH assay.

Manganese dioxide (MnO_2_) NPs and V_2_O_5_ nanowires were assembled through a pDA linker and exploited for mimicking the intracellular GPx, SOD, and CAT enzyme-based defense mechanism through a synergistic antioxidative effect [112]. Both in vitro and in vivo experiments showed that the nanocomposite is highly biocompatible, and possesses an excellent intracellular ROS removal ability to protect cell components against oxidative stress. Prasad et al. engineered a multifunctional system comprised of a polyelectrolyte–albumin complex and MnO_2_ NPs that were able to increase tumor oxygenation by 45% while increasing tumor pH from 6.7 to 7.2 in mice [113]. Simultaneous treatment of the murine breast tumor with NPs and ionizing radiation significantly inhibited tumor growth, because of the synergistic effect as compared to the radiation therapy alone. MnO_2_ and EGCG were both encapsulated into PLGA-HA copolymer generating stimuli-responsive particles [114]. The MnO_2_ nanocatalyst allowed the breakdown of H_2_O_2_ into oxygen gas, which augmented the interior pressure, thus accelerating the release of the EGCG, deriving a higher metabolic activity and more elevated secretion of pro-angiogenic factor in vitro in stem cells.

Cerium is a rare earth element, and in particular its oxide (CeO_2_) NPs are well-known for being an excellent catalyst and a material with strong antioxidant properties because of the capacity of its surface atoms to reversibly bind oxygen by switching between Ce^+3^ and Ce^+4^ oxidation states [115,116]. Recently, Soh and colleagues improved the scavenging capacity by preparing mixed ceria-zirconia NPs that were shown to be able to reduce mortality and systemic inflammation in vivo in a sepsis mice model [117]. Pu et al. conducted a systematic study on gold (Au) NPs synthesized by using chitosan with a diverse degree of deacetylation and molecular weight as a stabilizer and reducing agent, and then measured the corresponding radical scavenging ability by OH, DPPH, ABTS, and FRAP assays [118]. As expected, low MW chitosan (47.8 kDa) showed the highest antioxidant activity, which was also dependent on the size, shape, and concentration of the NPs, with the spherical ones showing higher activity than irregular or polygonal ones.

Chitosan and antioxidant molecules were also attached to magnetic nanomaterials through a layer-by-layer technique in order to exploit the properties of both components [119]. In particular, maghemite (γ-Fe_2_O_3_) NPs were first coated with (anionic) heparin, and then with (cationic) chitosan, which had been previously functionalized with various phenolic compounds, including gallic acid (CS-GA), hydroquinone (CS-H), and phloroglucinol (CS-P). Among the free polymers, the highest antioxidant activity was observed with the CS-GA, but no significant differences could be observed when radical scavenging comparison was performed with the polymer-coated NPs. Similarly, while an external magnetic field was able to increase internalization of the iron oxide NPs without antioxidants, it did not affect the internalization of those functionalized with the phenols, suggesting that the small antioxidant molecules might facilitate uptake to a plateau level.

## 3. Conclusions

Oxidative stress is at the basis of several pathologies, and its reduction can help to control them. In this regard, biocompatible antioxidant polymers have attracted much interest, because they are relatively cheap and easy to functionalize. Nevertheless, different polymeric formulations can lead to different properties, and thus to different applications in the biomedical field. Natural extracts are relatively cheap and have an extremely low risk of negative side effects. On the other hand, their characteristics are largely influenced by the source and the extraction method employed; this is one of the main reasons why they are somewhat criticized, and have difficulties when finding clinical applications. Polymeric films and hydrogels have been widely exploited for the topical treatment of inflammations and wound healing, while novel nanotechnological approaches allowed the entrapment and protection of antioxidants before delivery to the target site, as well as a combination of free radical scavenging abilities with other properties. Specific targeting is possible by functionalizing the surface of the NPs with ligands that are able to interact with overexpressed receptors, for example, on the surface of tumor cells, or on the BBB. In addition, the release con be triggered by a precise biological component, such as an enzyme, or event, such as reaching a region with a particular pH (i.e., acidic) or a high amount of free radicals.

The use of natural antioxidants as drug active compounds is rapidly growing, especially in cancer, neurodegenerative, and inflammatory diseases in which the redox homeostasis is compromised, dramatically affecting the development and progression of the pathologies [120]. Thus, the attempt to reestablish this imbalance is a fundamental component of several therapeutic approaches. Nevertheless, the advent of new polymeric antioxidant formulations, such as films, hydrogels, and nanoparticles, opens the debate on their efficacy/long-term exposure relationship and poses questions about dosing, time of administration, and pharmacology, as many of these formulations are relatively unknown to current regulatory guidelines (https://www.ema.europa.eu/en/excipients-dossier-application-marketing-authorisation-medicinal-product). This is the case of nanoparticles that can be composed of or loaded with active components exhibiting RONS scavenging properties: so far, a limited number of in vivo studies have provided sufficient evidence of efficacy in addition to the analysis of their safety and the potential effects of their degradation byproducts [121]. The lack of a comprehensive and thorough depiction of the biological challenges related to the biomedical development of safe and effective antioxidants demonstrates that many hurdles need to be overcome. In this sense, a review that summarizes the recent findings on the preparation of polymeric formulations for the reduction of the oxidative stress may contribute to defining which are the common functional features that an ideal antioxidant should possess. Nevertheless, the authors believe that the studies here presented clearly demonstrate that the polymeric formulations hold great potential in the reduction of the oxidative stress in clinical practice.

## Figures and Tables

**Table 1:** Summary of the antioxidant polymers deriving from natural sources and their effects. ABTS: 2,2′-azino-bis(3-ethylbenzothiazoline-6-sulfonic acid), DPPH: 2,2-diphenyl-1-picrylhydrazyl, MW: molecular weight.

Source Plant	Antioxidant	Results	Ref.
*Astragalus membranaceus* and *Glycyrrhiza uralensis*	phenols and flavonoids	higher antioxidant capacity in vitro than the theoretical sum of two individual herbs, probably because of a synergistic effect	[21]
*Phyllanthus Emblica*	polysaccharides and phenols	antioxidant capacities comparable to BHA and BHT antioxidants proportionally to the phenol content	[22]
*Phyllanthus Emblica*	low MW hydrolysable tannins (emblicanin-A, emblicanin-B, punigluconin, and pedunculagin)	significant decrease of several CVD risk factors	[23]
*Acanthophyllum acerosum* roots	polysaccharide (20.8% d-glucose, 66.2% d-galactose, and 13.0% l-arabinose)	able to scavenge DPPH radicals, but lower activity compared to that of ascorbic acid at the same concentration	[24]
*Epimedium acuminatum*	polysaccharides	higher antioxidant activities by hot water extraction apparently because of a more regular and smoother surface	[26]
*Pharbitis nil* seeds	polysaccharides, with minor percentages of uronic acid and proteins	remarkable ABTS and DPPH radical scavenging activities by ultrasonication extraction	[28]
white hyacinth bean	polysaccharides	lower scavenging activity compared to ascorbic acid, but ability to stimulate the growth of several probiotics	[29]
barley	β-glucan (mostly β-(1,3-1,4)-d-glucan)	antioxidant activity higher than that of several polymers used as food additives, supposedly because the β-glucan decreased the number of pro-inflammatory cytokines (mostly IL-6 and tumor necrosis factor-alpha, TNF-α) and increased that of the antioxidants	[31]
*Cystoseira barbata*	laminaran polysaccharide	noticeable antimicrobial and antioxidant properties in vitro, as well as wound-healing promotion in vivo	[32]
*Spirulina sp*	apo-c-PC β subunit	antioxidant activity lower than that of the natural extract, confirming the scavenging role of the bilin chromophore	[36]
*Spirulina subsalsa*	PCB-CpcB(C-82) fluorescent phycocyanin β subunit	stronger hydroxyl and DPPH free radicals scavenging activity than apo-CpcB, probably because of the bilin binding	[37]
*Spirulina platensis*	c-PC	reduced apoptosis, DNA damage, and autophagy in oxidatively stressed blastocysts of porcine embryos	[38]
commercially available *Spirulina* powders	carotenoids and c-PC	the c-PC extract had a stronger antioxidant activity compared to the carotenoid fraction	[39]

**Table 2:** Summary of the antioxidant polymeric films and their effects.

Base Polymer	Additional Antioxidant	Results	Ref.
chitosan	aqueous green tea extract	improved mechanical, water vapor barrier, and antioxidant properties	[43]
chitosan	montmorillonite and pomegranate rind powder extract	enhanced water vapor permeability and mechanical properties and excellent antioxidant activities	[44]
chitosan	carvacrol and pomegranate peel extract	decreased transparency but improved antioxidant activity	[45]
chitosan	caffeic acid or gallic acid	better pH-dependent antioxidant and antimicrobial properties	[46]
chitosan	α-tocopherol	improved UV protection, higher water vapor permeability, and better antioxidant capacity	[47]
chitosan	rosemary, ginger, sage, tea tree, and thyme essential oil (EO); ginger, rosemary, sage, black tea, green tea, and kenaf leaves HAE	improved light barrier and tensile strength	[48]
chitosan	rosemary, ginger, sage, tea tree, and thyme EO; ginger, rosemary, sage, black tea, green tea, and kenaf leaves HAE	the highest diffusion and antioxidant activity for the films with ginger, sage, or rosemary EO	[49]
chitosan and inulin	oregano and thyme EO	better physicochemical properties and improved antioxidant and antimicrobial activity	[50]
reacetylated chitosan	annatto powder and vitamin C	significantly improved ROS scavenging ability	[52]
chitosan and wheat starch	citric acid, α-tocopherol, thyme and basil EO	films containing α-tocopherol showed a higher antioxidant ability without affecting the mechanical properties	[54]
starch	cocoa nibs extract (CNE)	ability to quench 100% of ABTS and 94% of DPPH produced radicals with the 1% CNE-containing film	[55]
polyurethane	lignin fractions	reproducible method for obtaining homogeneous lignin products with reliable physicochemical properties	[57]
cellulose nanofibrils	tannin extract	improved antioxidant and UV-adsorbing properties	[58]
carboxymethyl cellulose	sodium alginate (SA) and epigallocatechin gallate (EGCG)	edible EGCG-releasing films with strong antioxidant activity in fatty foods	[59]
polyvinyl alcohol (PVA)	tannin	good antioxidant activities	[60]
polylactic acid (PLA)	ferulic acid (FA), vanillic acid (VA), vitamin E (VE), and quercetin (Q)	highest antioxidant properties when FA and Q were combined at low concentration with PLA	[61]
gelatin	rosmarinic acid	excellent ultraviolet barrier capacity, good antioxidant properties, long-term antibacterial activity	[62]
gelatin	citric acid and chitosan	allowed the healing process in ex vivo assay in human skin	[63]
soybean protein isolate	cortex *Phellodendron* extract	good rheological properties and additional antioxidant and antimicrobial properties	[64]

**Table 3:** Summary of the antioxidant polymeric hydrogels and their effects. ROS: reactive oxygen species.

Base Polymer	Additional Antioxidant	Results	Ref.
PAbAE	curcumin and quercetin	controlled degradation rate and the degradation products suppressed the induced oxidative stress in HUVEC cells	[66]
PAbAE	curcumin and quercetin (25–38 wt% loading)	slow release of the antioxidant and inhibition of the oxidative stress	[67]
PAbAE	curcumin	ability to protect cells from radicals and increased tolerance to curcumin cytotoxicity	[68]
PAbAE	cystamine	environmental redox sensitivity and increased IC_50_ by an order of magnitude	[69]
poly(ethylene glycol)-*co*-poly(glycerol sebacate)	quaternized chitosan-g-polyaniline	good self-healing, free radical scavenging ability, antibacterial, and antioxidant activities for cutaneous wound healing; enhanced in vivo wound healing process	[70]
*N*-carboxyethyl chitosan	hyaluronic acid-graft-aniline tetramer	high free radical scavenging capacity, high swelling ratio and antimicrobial property; accelerated in vivo healing process	[71]
trimethylolpropane triglycidyl ether	tannic acid	good antioxidant ability at slightly acidic pH; robust antimicrobial property	[72]
hyaluronic acid (HA)	tannic acid	improved resistance to enzymatic degradation and antioxidant capacity	[73]
chitosan	gallic acid and dopamine	stronger antioxidant capacity in the GA-functionalized hydrogels and in those with longer chitosan chains	[74,75]
PPS-*b*-PDMA-*b*-PNIPAAM		ROS-triggered degradation and drug release	[76]
PPS-*b*-PDMA-*b*-PNIPAAM		in vivo differential release kinetics according to the specific degradation mechanism	[77]
methoxy poly(ethylene glycol)-poly(l-methionine)		accelerated release under oxidative stress conditions, both in vitro and in vivo	[78]
alginate	cerium oxide NPs	dose-dependent protection to beta cells from superoxide exposure	[80]

**Table 4:** Summary of the polymeric antioxidant NPs and their effects. RONS: reactive oxygen and nitrogen species.

Base Polymer	Additional Drug	Results	Ref.
melanin		free radical scavenging capacity similar to that of ascorbic acid in HeLa cells	[85]
polydopamine (pDA)		reduced ROS levels in vivo in murine macrophages; alleviated acute peritonitis and acute lung injury inflammation in murine models	[86]
pDA-coated hemoglobin		reduced the intracellular oxidative stress without affecting the blood constituents	[87]
polydopamine		reduced inflammation by subgingival injection in a murine periodontitis model	[88]
polydopamine		demonstrated activity against multiple RONS; reduced oxidative stress in a rat model of ischemic stroke	[89]
PEG-polymaleic acid (PMA)	dopamine	targeted dopamine delivery through the GLUT-1 transporter	[90]
HBA-HPOX		reduced expression of pro-inflammatory mediators in a murine model of asthma	[91]
PVAX	dexamethasone	reduced oxidative stress and suppression of the expression of TNF-a and iNOS in the lung of asthmatic mice	[92]
TA	CAT	inhibition of the oxidative stress and prevention of the expression of MMP-3, disintegrin, and ADAMTS-5 in an in vitro inflammation model of degenerative disc disease	[93]
vinylimidazole and vinylpyrrolidone	methacrylic derivatives of ibuprofen, α-tocopherol and α-tocopheryl succinate; dexamethasone	lower cisplatin-induced toxicity, downregulation of caspase 3/7 expression, lower IL-1β release, and intracellular ROS accumulation in vitro; reduced hearing loss in vivo	[94]
chitosan	dopamine	significant reduction of the oxidative stress in SHSY-5Y cells; increased enzymatic activity of both GPx and SOD	[95]
chitosan	genistein	efficient drug delivery to the brain after permeation through the nasal mucosa	[96]
chitosan	curcumin, quercetin, aspirin	synergistic effect in inhibiting colon cancer progression in HCT-116 cells	[97]
PCL	curcumin and resveratrol	sustained drug release, facilitated skin absorption, deeper penetration of resveratrol	[98]
PVP-b-PCL	resveratrol and DAP5	decreased production of pro-inflammatory cytokines and attenuated renal ischemia reperfusion (I/R) injury in vivo	[99]
PEG	resveratrol	optimal protection against oxidative stress in an ex vivo human erythrocytes-based model	[100]
Eudragit S100	curcumin	good ABTS antioxidant activity; inhibition of the drug release until degradation of the NPs	[101]
PLGA	curcumin	induced neural stem cells proliferation and neuronal differentiation in adult rats	[102]
curcumin		DPPH scavenging efficiency almost comparable to that of ascorbic acid	[103]
PCL	*Syzygium cumini* seeds extract	high protection against oxidized LDL particles in vitro	[104]
PCL	*Ilex paraguariensis* extract	significant reduction of chlorogenic acid permeated through the skin; increased topical antioxidant effect	[105]
graphene-like		reaction with hydroxyl radicals in macrophages	[106,107]
POM	molybdenum NPs	reduction of the clinical symptoms in mice affected by acute kidney injury	[108]
selenocysteine-derived		reduced oxidative stress in MDA-MB-231 cells	[109]
low MW chitosan-coated selenium		efficiently penetrated mice tissues and protected GPx activity	[110]
*Z. Officinale* root extract		good antimicrobial activity and excellent radical scavenging activity when compared to that of ascorbic acid	[111]
pDA	MnO_2_ NPs and V_2_O_5_ nanowires	excellent intracellular ROS removal ability both in vitro and in vivo	[112]
Polyelectrolyte–albumin complex	MnO_2_ NPs	increased tumor oxygenation by 45% in mice	[113]
PLGA-HA	MnO_2_ and EGCG	higher metabolic activity and more elevated secretion of pro-angiogenic factor in vitro in stem cells	[114]
Phospholipid–PEG	ceria-zirconia NPs	reduce mortality and systemic inflammation in vivo in sepsis mice model	[117]
chitosan	Au NPs	good antioxidant activity which was dependent on the size, shape, and concentration of the NPs	[118]
heparin, chitosan + GA/hydroquinone/phloroglucinol	maghemite NPs	highest antioxidant activity observed with CS-GA; an external magnetic field did not increase internalization of the NPs functionalized with the phenols	[119]
